# Longitudinal Study of Oral Microbiome Variation in Twins

**DOI:** 10.1038/s41598-020-64747-1

**Published:** 2020-05-14

**Authors:** Marcelo Freire, Ahmed Moustafa, Derek M. Harkins, Manolito G. Torralba, Yun Zhang, Pamela Leong, Richard Saffery, Michelle Bockmann, Claire Kuelbs, Toby Hughes, Jeffrey M. Craig, Karen E. Nelson

**Affiliations:** 1 Departments of Human Biology and Genomic Medicine, J. Craig Venter Institute, La Jolla, CA 92037 USA; 2 Department of Biology, The American University in Cairo, New Cairo, 11835 Egypt; 3 Departments of Human Biology and Genomic Medicine, J. Craig Venter Institute, Rockville, MD 20850 USA; 4 IMPACT Strategic Research Centre, Deakin University School of Medicine, Geelong, VIC 3220 Australia; 5 Adelaide Dental School, The University of Adelaide Epigenetics, Murdoch Children’s Research Institute and Department of Paediatrics, The University of Melbourne, Parkville, 3052 Victoria Australia

**Keywords:** Microbiome, Predictive markers, Dental caries

## Abstract

Humans are host to a multitude of microorganisms that rapidly populate the body at birth, subject to a complex interplay that is dependent on host genetics, lifestyle, and environment. The host-associated microbiome, including the oral microbiome, presents itself in a complex ecosystem important to health and disease. As the most common chronic disease globally, dental caries is induced by host-microbial dysbiosis in children and adults. Multiple biological and environmental factors are likely to impact disease predisposition, onset, progression, and severity, yet longitudinal studies able to capture these influences are missing. To investigate how host genetics and environment influenced the oral microbial communities over time, we profiled supragingival plaque microbiomes of dizygotic and monozygotic twins during 3 visits over 12-months. Dental plaque DNA samples were amplified by targeting the 16S rRNA gene V4 region, and microbial findings were correlated with clinical, diet and genetic metadata. We observed that the oral microbiome variances were shaped primarily by the environment when compared to host genetics. Among the environmental factors shaping microbial changes of our subjects, significant metadata included age of the subject, and the age by which subjects initiated brushing habits, and the types of actions post-brushing. Relevant heritability of the microbiome included *Actinomyces* and *Capnocytophaga* in monozygotic twins and *Kingella* in dizygotic twins. *Corynebacterium* and *Veillonella* abundances were associated with age, whereas *Aggregatibacter* was associated with younger subjects. *Streptococcus* abundance showed an inverse association over time, and *Selenomonas* abundances increased with brushing frequency per day. Unraveling the exact biological mechanisms in caries has the potential to reveal novel host-microbial biomarkers, pathways, and targets important to effective preventive measures, and early disease control in children.

## Introduction

Across various human body habitats, including the oral cavity, mutually beneficial relationships between the host and its microbiome lead to homeostatic phenotypic patterns. While shifts in gastrointestinal^1,2^ and skin^3–5^ habitats have advanced our understanding of host-microbial dysbiosis, the oral counterpart has not received the same level of mechanistic interrogation, particularly with respect to host genetics link. One central question remaining unanswered is the extent to which differences in the microbiome are explained by host genetics. As one of the most diverse microbial ecologies in humans, the oral microbiome has critical importance to local oral and systemic health. In fact, oral microbial dysbiosis underlies many human oral diseases with systemic consequences, but the exact mechanism regulating microbial patterns associated with health to disease transition and from acute to chronic lesions remains elusive. Loss of host-microbial homeostasis in the oral habitat leads to dysbiosis and a range of oral and systemic conditions, including caries, periodontal disease, and cancers. Oral diseases such as caries present an extensive economic burden in the United States^6^, whose national costs exceeds $100 billion annually^7^. For untreated tooth decay, it is estimated that more than 2 billion cases exist worldwide with an alarming incidence of 190 million new cases each year, making the study of microbial and genetic forces in caries etiology a topic important to public health^8^.

In addition to the above, caries ecology is more complex than a monogenic species model can detail. Caries is a descriptor of an effective dynamic process rather than a disease. In the early stages, for example, it is reversible, but it becomes irreversible in late stages. More than one phenotype, including relevant host-microbial interactions and environmental changes throughout life, modulate the severity of caries. Although correlative studies point to the genera *Streptococcus* and *Lactobacillus* as main etiological factors, they do not capture the entire ecological complexity of the oral microbiota. Host-microbial interactions and environmental stimuli (e.g., socioeconomic status, diet, hygiene, and others) however, are essential to interpreting clinically relevant phenotypes. Besides the impact of the oral microbiome on its immediate local environment in the oral cavity, systemic sites are also impacted. Oral-systemic connections include immunological, neurological, metabolic, respiratory, cardiovascular, digestive and broader systemic health^8,9^. Thus, a deeper understanding of the molecular aspects of caries will contribute to the development of molecular predictors that may facilitate diagnostics, and therapy while providing feedback for therapeutic applications.

In an earlier cross-sectional investigation by our group, we investigated the relationship between the oral microbiome, host-associated microbial communities, host genetics, and environmental factors in the caries phenotypes in 485 dizygotic and monozygotic twins (241 twin pairs and one set of triplets) aged 5–11 at a single visit^10,11^. Our previous studies have identified several heritable bacterial taxa relevant to enamel and dentin caries. We have found that there was no specific association between caries with streptococci or other associated taxa. Also, genetics could not explain the phenotypes of twins when we surveyed in one cross-sectional time point^10–12^. Caries pathology was shown to be more complex than changes one pathobiont could explain.

Herein, we have followed up on our monozygotic (MZ) and dizygotic (DZ) twin study for a 12-month period and sampled 143 of the twin pairs at three visits, approximately six months apart. We have evaluated the extent that caries had advanced through the enamel and penetrated into the dentin or just remained confined within the enamel clinically. In this longitudinal analysis, we evaluated the supragingival microbiome found in plaque (biofilm) from twin pairs with or without carious lesions. We sought to further explore which oral bacterial taxa were associated with health and disease, and which taxa were genetically or environmentally driven. Among the three visits, significant metadata that shaped the microbiome included the age of the subject, the age at which subjects initiated brushing habits, and the actions post-brushing. We observed that the most heritable taxa were associated with the subject brushing habits and age.

## Results

**Figure 1:**
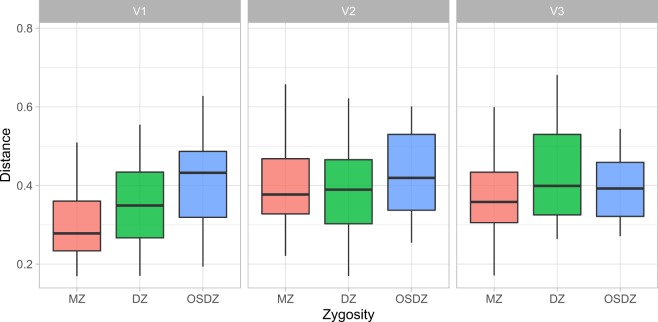
Oral microbiome distance by relationship and visit. Dissimilarity on the y-axis was estimated using the Bray–Curtis index. The relationships tested were MZ: monozygotic twins, DZ: dizygotic twins, OSDZ: opposite-sex dizygotic twins. The visits are displayed as V1: visit #1, V2: visit #2, and V3: visit #3. Significance was tested through ANOVA and p-values include 0.0000928 for V1, 0.1232370 for V2, and 0.5195040 for V3. Adjusted pairwise p-value of statistical significance between the distances: MZ vs DZ = 0.07, MZ versus OSDZ = 0.00053, DZ vs. OSDZ = 0.077.

**Figure 2:**
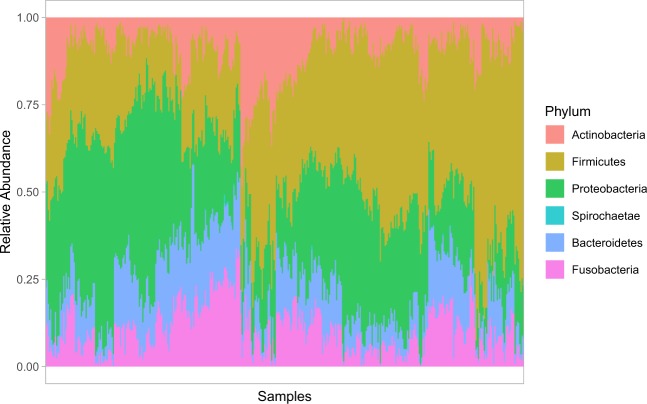
Major bacterial phyla of the oral microbiome. Bar plot shows the relative abundance of the major bacterial phyla (phyla with relative abundance ≧ 1% in any sample).

In this study, the ecological associations of supragingival microbial taxa were most affected by environmental factors such as age over the time. Microbiome data from oral plaque swabs from 143 twins (70 twin pairs, and 1 set of triplets) with 62 monozygotes and 81 dizygotes (36 opposite sex dizygotes, and 45 same sex dizygotes) were surveyed. Eligible individuals were sampled 3 times at approximately 6 months intervals. The age at diagnosis ranged from 5.5–12 years old with a median of 9 years old. Microbial dissimilarity was estimated by Bray–Curtis index, and the results showed that differences initially influenced by inherited genetic backgrounds reduced over time from visit 1 to visit 3 (Fig. 1). Pairwise comparisons within each visit demonstrated that dissimilarity significance decreases over time (Table 1). Various bacterial communities composed of operational taxonomic units (OTUs) were detected for visit one (468), visit two (362), and visit three (343), with a total of 540 unique OTUs across the three visits, from 16 phyla with five major phyla (median abundance > 0.01); Firmicutes (0.330), Proteobacteria (0.294), Bacteroidetes (0.125), Fusobacteria (0.103), Actinobacteria (0.0762) (Fig. 2).

**Table 1:** Pairwise comparisons within each visit. P-values for pairwise comparisons within each visit from Fig. 1 were listed.

-y-	group 1	group 2	p	p.adj	p.format	p.signif
**Visit 1**						
dist	MZ	DZ	0.0719831	0.07200	0.0720	ns
dist	MZ	OSDZ	0.0000489	0.00015	4.9 e0.5	****
dist	MZ	Unrelated	0.0000000	0.00000	<2e-16	****
dist	DZ	OSDZ	0.0072112	0.01400	0.0072	**
dist	DZ	Unrelated	0.0000000	0.00000	1.5e-14	****
dist	OSDZ	Unrelated	0.0000308	0.00012	3.0e-05	****
**Visit 2**						
dist	MZ	DZ	0.9812185	9.8e-01	0.981	ns
dist	MZ	OSDZ	0.1420208	2.8e-01	0.142	ns
dist	MZ	Unrelated	0.0000000	0.0e + 00	5.1e-09	****
dist	DZ	OSDZ	0.0622522	1.9e-01	0.062	ns
dist	DZ	Unrelated	0.0000000	2.0e-07	4.5e-08	****
dist	OSDZ	Unrelated	0.0059690	2.4e-02	0.006	**
**Visit 3**						
dist	MZ	DZ	0.1710755	5.1e-01	0.17108	ns
dist	MZ	OSDZ	0.4075724	8.2e-01	0.40757	ns
dist	MZ	Unrelated	0.0000000	2.0e-07	2.6e-08	****
dist	DZ	OSDZ	0.4574852	8.2e-01	0.45749	ns
dist	DZ	Unrelated	0.0003419	1.4e-03	0.00034	***
dist	OSDZ	Unrelated	0.0000066	3.3e-05	6.6e-06	****

**Figure 3:**
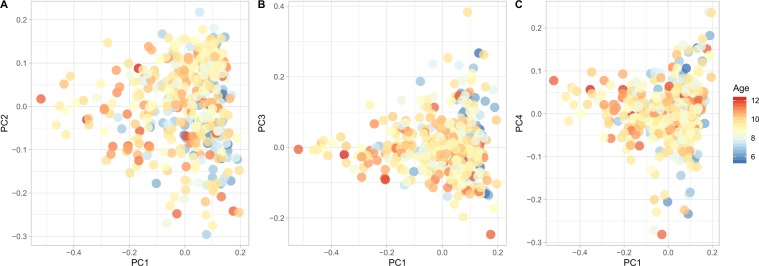
Principal component analysis of the oral microbiome. (**A**) PC2 vs. PC1, (**B**) PC3 vs. PC1, (**C**) PC4 vs. PC1. In each panel, the age of the twin is color-coded.

**Figure 4:**
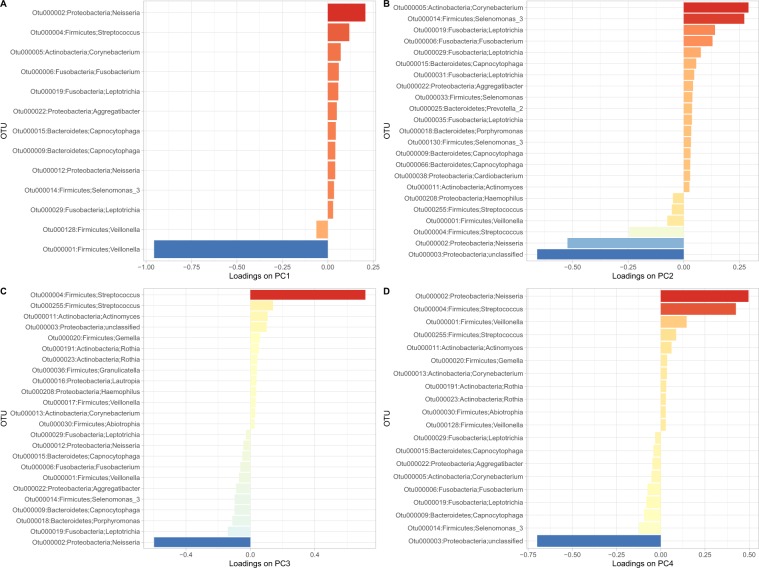
Driver bacterial species of the principal components of the oral microbiome. The major bacterial species contributing to the principal components PC1, PC2, PC3, and PC4 are indicated on the y-axis in (**A–D**), respectively. The contribution of the OTUs to the principal components (PC) of the microbiome is represented by the loadings of the OTUs on the PCs on the x-axis. The OTUs were ranked according to their loadings, which is also color-coded.

We employed principal component analysis (PCA) to analyze the effect of the microbiome, host genetic, and age contributions to oral health and caries phenotypes. Overall, the microbiome variance of all the samples was explained by four principal components (PCs; Fig. S1). No obvious clusters were found, but tendencies of clusters according to age. Samples from younger subjects (blue) tended to cluster on the positive side of PC1, compared to samples of older ages (red) on the negative side of PC1 (Figs. 3, 4). Specific increase microbial abundances in each group included the Firmicutes *Veillonella* and the Bacteroidetes *Capnocytophaga* (Fig. S2).

At the species (OTU) level, most variance was explained by species within the phyla including, Proteobacteria (*Neisseria mucosa*), Firmicutes (*Streptococcus*, *Veillonella*, Actinobacteria (*Corynebacterium)*, Bacteroidetes (Capnocytophaga), Fusobacteria (*Fusobacterium*, *Leptotrichia)* (Fig. 4). OTUs that were positively associated with the presence of caries were also impacted by environmental factors and showed positive co-existence with another taxon. For example, *Corynebacterium* which is known for healthy oral and nasal mucosa functions^13^, was part of a highly connected interactivity with *Neisseria*, the genera associated with environmental variation and oral health. *Streptococcus*, a taxon associated with high prevalence of caries showed inverse relationships with unclassified *Veillonella* (Fig. 4).

**Figure 5:**
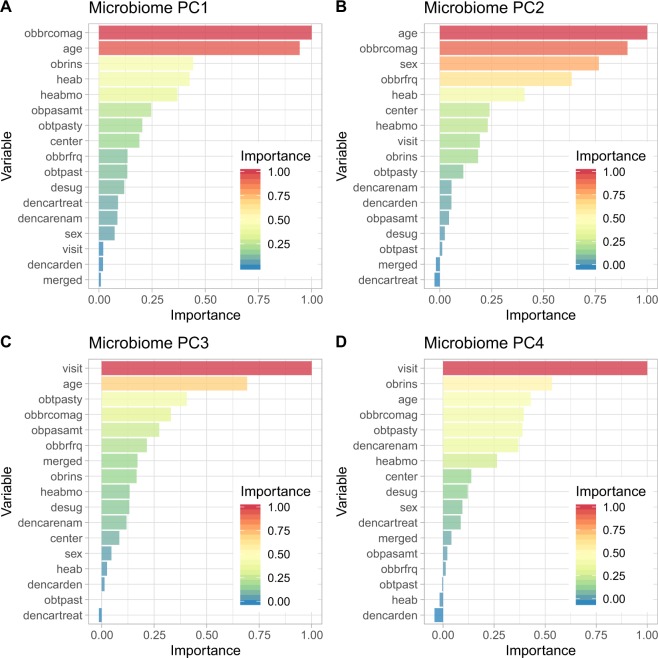
Importance of the environmental metadata towards oral microbial principal components. The levels were normalized to the largest value (scale 0–1) and displayed as PC1-PC4 (A-D). The contribution of the OTUs to the principal components (PC) of the microbiome is represented by the loading of the OTUs on the PCs. The OTUs were ranked according to their loadings, which is also color-coded. Panels A to D correspond to PCs 1–4, respectively. *Please see* Supplementary Table 1 *for further details*.

**Figure 6:**
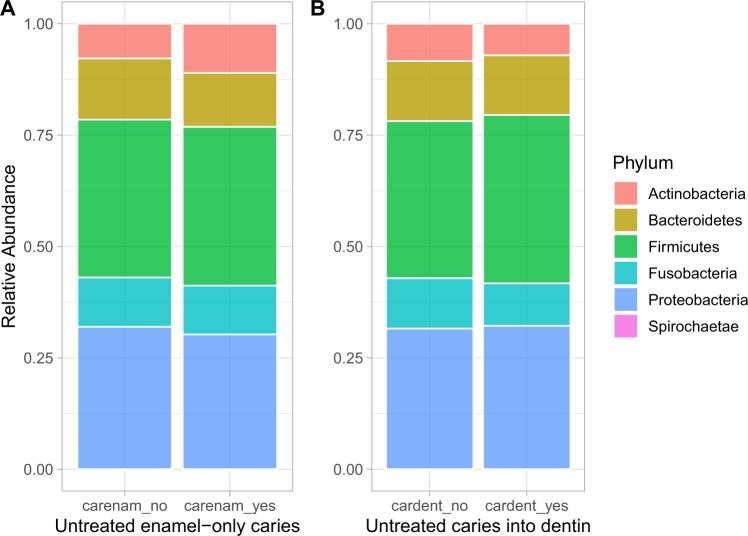
Caries effect of bacterial phyla distribution. (**A**) Bacterial phyla with or without untreated enamel caries. (**B**) Bacterial phyla with or without untreated caries in dentin.

Clinically, the major parameters contributing to PC1 and PC2 were the subject age and the age at which subjects began brushing their teeth (obbrcomag) (Figs. 5A,B, 6S). The main factors contributing to PC3 and PC4 were brushing frequency and actions post-brushing including expectoration and rinsing, expectoration only and no rinsing, and swallowing (obrins) (Figs. 5C,D, 6S).

Statistical analysis of sample variance from the three visits indicated significant abundance differences of five species belonging to the following phyla: Firmicutes, Actinobacteria, Bacteroidetes, Fusobacteria, Proteobacteria and p-values were indicated in Fig. S3. Firmicutes (*Veillonella*) was the most significant species influenced by age (Fig. S2). Proteobacteria (*Neisseria*) was the most significant species affected by the age at which the subjects began brushing (Fig. S4) (stratified into 9 categories Supplementary Table 1). Whereas, Firmicutes (*Veillonella* and *Streptococcus*) were mostly influenced by visit and whether they expectorated and/or rinsed after brushing (obrins) (Fig. S5). Previously reported caries-associated species (*Streptococcus mutans, Lactobacillus*) were either undetected or in low abundances from the first visit to final visit. Taxa increasing with the subjects’ age included *Veillonella* and *Corynebacterium*, while *Aggregatibacter* abundance was directly correlated with younger age (Supplementary Fig. 2). Abundance levels of *Streptococcus* decreased according to brushing frequency, whereas *Selenomonas* showed increasing abundance with visit numbers.

**Figure 7:**
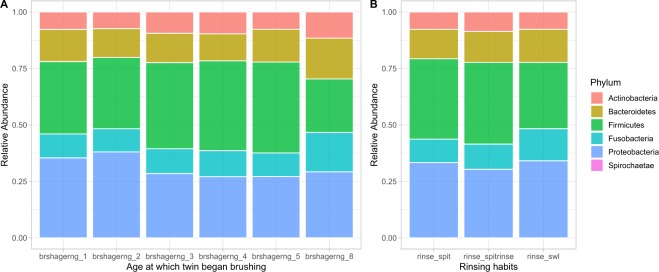
Behavioral effect on abundance of oral microbiome phyla. (**A**) Phyla abundance against the age of starting brushing (Brshagerng_1; <6 months of age, brshagerng_2; 6–12 months of age, brshagerng_3; 1–1.5 years of age, brshagerng_4; 1.5–2 years of age, brshagerng_5; 2–2.5 years of age, brshagerng_8; 5–6 years of age). (**B**) Phyla abundance against the rinsing habits (spit and rinse; rinse_spit, rinse; spit and no rinse; rinse_spit, swallow; rinse_swl). *Please see* Supplementary Table 1 *for further details*.

In the longitudinal study, we also evaluated the influence of caries type (enamel and dentin) and severity to microbiome changes. Microbial diversity was also confirmed according to the genetic background. The marginally significant p-value (0.098 for MZ and enamel caries) suggests a decreased Shannon diversity for those subjects who had caries in enamel (Supplementary Fig. 7), revealing that overall diversity is not shaped by types of lesions and genetic backgrounds. A comprehensive phyla distribution according to the type of lesions (Fig. 6) and age of brushing and rising habits (Fig. 7). These data also showed that specific taxa associated with healthy and caries dentition covaried with age, brushing habits and exhibited distinct ecological interactions with these environmental factors (Fig. 7). Dissimilarity estimated using the Bray–Curtis index (Fig. 1), showed that these environmental forces are controlling the microbiome through time (Supplementary Figs. 5 and 6). Thus, potentially, taxa from cariogenic may increase as subjects transition to an older age while being exposed to continuous environmental factors, including distinct brushing habits. Overall, environmental forces were observed to be the driving forces governing microbiome changes prior to enamel and deeper dentin caries formation.

## Discussion

Caries is the most prevalent multifactorial infectious disease worldwide^14^. Relevant host genetic components influencing salivary flow, tooth anatomy, and host immune response account for 40–60% of disease initiation^15,16^, yet the exact specific contributing factors remain elusive. While recent results indicate the initial impact of the host and the prevalence of caries^17^, very little has been learned in regards to the heritability of the oral microbiome. To dissect the role of host genetics influencing the oral microbiome of children with and without dental caries, we investigated 429 data points from 143 dizygotic and monozygotic twins over 3 visits. Dental plaque samples were collected before the examination of all visits from participants at the University of Adelaide Craniofacial Biology Research Group Tooth Emergence and Oral Health Study and the Murdoch Children’s Research Institute, Peri/Postnatal Epigenetic Twins Study^10^. The results presented here and previously add to existing evidence of environmental regulation on the human microbiome.

In a cross-sectional setting, we have previously observed that the caries phenotype was statistically significant correlated with specific changes in microbial profiles including *Streptococcus*^11^. Here, we provide longitudinal evidence that the composition of the human oral microbiome and caries phenotype are mostly influenced by acquired behavior and not by genetics. In contrast to our biofilm analysis, salivary sampling of 752 twin pairs demonstrated that a number of microbial phenotypes were more than 50% heritable^17^. The heritable microbiome in our study was clearly influenced by environmental changes through the 3 visits and as the subjects matured with the individualized environment, newly acquired behavior shapes microbiome changes.

Evidence that the human microbiome is shaped by host genetics has been much less investigated for the oral compartment when compared to the gut, mainly in the context of phenotypic predictions of health and disease states. Earlier reports were focused mainly on the gut dysbiosis, and have elucidated that the heritable microbiome was influenced by changes in nutrition^18^ and gut mucosa host response^19^. Extensive research has previously shown that heritable traits from body metabolic functions^20^ and host loci were associated with specific taxa, including *Akkermansia* and *Bifidobacterium*. The oral tissues are composed of unique soft epithelial mucosa, and mineralized tissues, including enamel, dentin and cementum and alveolar bone. A large-scale genome-wide association study (GWAS) in addition to a multi-cohort meta-analysis recently suggested 29 loci associated with dental caries in adults^21^. Among children, small GWAS studies examined caries in the primary dentition and nominated 7 genes, 2 of which showed additional evidence of association in follow-up studies among children and adults^22^. Thus, these host factors are shaping how the microbiome is able to transition to pathogenicity. The enamel is the hardest tissue of the human body and prone to demineralization by acid produced from bacteria and diet. When deeper enamel lesions occur, this allows the oral microbes to access vascularized space, the dentin, the dental pulp, and even the alveolar bone, reaching minor and major circulation and distant organs. Previous cross sectional twin-based studies on genetic heritability of oral microbes have presented distinct findings to our results^23–25^. It has been suggested that the levels of the pathobiont *Streptococcus. mutans* and its specific metabolic attributes such as acid production were highly heritable, while our findings suggest community-based variations were dependent on environmental factors and not genetics (Fig. 5).

The taxa that were positively associated with caries and covaried with age, were influenced by time during the study period. Distinct ecological interactions of taxa associated with phenotypic changes were observed according to habitual patterns. Clearly, specific OTUs that influenced the principal component analysis (Fig. 4) were shaped by age. PC1 was influenced by age with decreasing associations after visit 1. In contrast PC2 had an inverted relationship and was influenced by increased age. These observations raise new questions about chronic disease heritability, especially because of the influence from the environment to the microbiome composition. Taxa associated with normal flora including *Neisseria, Fusobacteria, Corynebacterium*, were more significantly affected by the genetic background, and four environmental factors, with no association for the type of caries (enamel or dentin).

Taxa associated with caries in this study have been associated with other conditions and mucosal diseases. *Corynebacterium*, for example, was previously associated with healthy oral and nasal mucosa and shown to be highly connected with *Neisseria*, a genus associated with oral health^26^. While a physiological flora consists a high percentage of streptococci, e.g. S. mitis, S. oralis, here we have found that the genus streptococcus was not associated to health or disease. An inverse relationship between levels of *Streptococcus* and unclassified *Veillonella* was found in visit 2 and 3. The observations presented here, in addition to significant heritability variation in the microbiome and environmental variations expressed over time support the hypothesis of ecological changes and community-based response. Future studies with mechanistic models are needed to validate how ecological factors of the microbiome can ultimately impact the host. *Actinomyces* and *Capnocytophaga* were significantly correlated in monozygotic twins, and *Kingella* showed positive correlations in dizygotic twins. Environmental factors were the most important factors in influencing dynamic changes in the oral microbiome which in turn impacted taxa coexistence, disease incidence, and severity.

Our findings indicate that the host environment drives changes in the oral microbiome over time. Consistently with our study on supra-gingival plaque, salivary microbial abundance was also influenced by the environment and host phenotypes^27^. While the biofilm is directly correlated to demineralization of enamel, saliva provides the source of microbes which through evolution adapted to attach to the tooth surface. Diet is also a factor that shapes the microbiome^28,29^, and, while diet was not part of this investigation, the role of dietary modulations in the oral microbiome metabolism and its impact to oral phenotypes need to be further investigated. The context of the individual’s environmental exposure offers an opportunity to predict risk for early disease markers, providing insights into the development of next generation diagnostics.

The data presented here, however, emphasized the notion that complex microbial interactions were mostly influenced by individual habits and age, and not genetic background. Future evidence of functional metabolism will allow deeper understanding of the exact genes, gene clusters and metabolic products responsible for influencing the functional microbial products that are shaping the ecological environment, which in turn are impacting the host response leading to health or disease. To investigate further the role that taxa play in disease pathogenesis, the microbiome and the host immune system must be further assessed concomitantly in higher resolution and functional surveys (metagenomics, metatranscriptomics, and metabolomics) with pipelines destined for the integration of molecular information in combination with detailed clinical input. These integrative approaches will lead to a precise map of the functional microbiome landscape and will help trace microbially-derived biomarkers important to homeostasis and dysbiosis.

## Methods

### Cohort description

Our objective was to compare longitudinal microbiome differences in supragingival dental plaque of children with and without dental caries. Dental plaque samples were collected from participants of the University of Adelaide Craniofacial Biology Research Group Tooth Emergence and Oral Health Study (CBRG) (n = 105), and the Murdoch Children’s Research Institute (MCRI) Peri/Postnatal Epigenetic Twins Study (PETS) (n = 38), as previously described^10,30^. Human research with PETS subjects was approved by the Royal Children’s hospital Human Research Ethic Committee (#3174), and the CBRG cohort was approved by The University of Adelaide Human Research Ethics Committee (#H-2013-097). Research at the J. Craig Venter Institute was approved by the JCVI Institutional Review Board (#2013-182). All research was performed according to the listed institutions guidelines and informed consent was obtained from all participants’ parent and/or legal guardians. Inclusion criteria included 5-11-year-old twins whose parents consented to this particular arm of the study. The cohort was comprised of 143 twins (70 twin pairs, and 1 set of triplets) comprise of 62 monozygotes and 81 dizygotes (36 opposite sex dizygotes, and 45 same sex dizygotes). Eligible individuals were sampled 3 times at approximately 6-month intervals throughout the course of 12 months. Supragingival plaque samples were obtained at the commencement of a dental examination. Prior to sample collection, participants were guided not to brush their teeth the night preceding the sample collection. Metadata were collected from three separate questionnaires completed by the parents during the period from consent to prior to the dental examination being undertaken. The clinical questionnaires consisted of a total of 132 questions to survey oral and medical health, dietary patterns, and development patterns, and dental hygiene.

Visual inspection of the oral cavity followed International Caries Detection and Assessment System (ICDAS II)^31^. The ICDAS II was used to assess and define dental caries at the initial and early enamel lesion stages through to dentin and more advanced stages of the disease. Examiners were experienced clinicians who had undergone rigorous calibration and were routinely recalibrated across measurement sites to minimize error. Caries history in each participant was initially reduced to a whole-mouth score and three classifications were utilized: no evidence of current or previous caries experience; evidence of current caries affecting the enamel layer only on one or more tooth surfaces; evidence of previous or current caries experience that has progressed through the enamel layer to involve the dentin on one or more tooth surfaces (including restorations or tooth extractions due to caries). For the purpose of phenotypic analysis, we classified disease states from twins as presence of caries in enamel or dentin. Twin pairs were selected for sequencing by examining ordination plots from the broader 16S rRNA gene sequencing study and then selecting: (1) twins of the same phenotype that were closely related; and (2) twins discordant for caries that were divergent in ordination space.

### Sample collection, DNA extraction, library prep and sequencing

Plaque sample collection and DNA extraction were as previously described^10^. Briefly, plaque samples were thawed at 4 °C and vortexed thoroughly. Samples were resuspended in 300 µl of TES buffer (20 mM Tris-HCl, pH 8.0, 2 mM EDTA, and 1.2% Triton X-100), pulse vortexed and incubated at 75 °C for 10 min followed by cooling to room temperature. Microbial cells were digested using lysozyme (200 mg/mL) and Proteinase K (40 ul at 20 mg/mL). DNA was extracted twice using phenol/chloroform isoamyl alcohol extraction and precipitated using ethanol. Precipitated DNA was centrifuged at 13,000 × g for 10 min and the DNA pellet was washed with 80% ethanol. After air drying, the DNA pellet was resuspended in TE buffer (20 mM Tris-HCl, pH 8.0; 1 mM Na-EDTA) and stored at −20 °C.

### 16S library preparation of plaque samples and sequencing

DNA extracted from plaque samples was amplified using primers that targeted the V4 region of the 16S rRNA gene^30,32^. These primers included the i5 and i7 adaptor sequences for Illumina MiSeq pyrosequencing as well as unique 8 bp indices incorporated onto both primers such that each sample receives its own unique barcode pair. This method of incorporating the adaptors and index sequences onto the primers at the PCR stage provided minimal loss of sequence data, generating sequence reads which are all in the same 5′-3′ orientation, when compared to previous methods that would ligate the adaptors to every amplicon after amplification (data not shown). Using approximately 100 ng of extracted DNA, the amplicons were generated with Platinum Taq polymerase (Life Technologies, Carlsbad, CA) using the following cycling conditions: 95 °C for 5 minutes for an initial denaturing step followed by 95 °C for 30 sec, 57 °C for 30 sec, 72 °C for 30 sec for a total of 35 cycles followed by a final extension step of 72 °C for 7 minutes then stored at 4 °C. The amplicons were purified using QIAquick PCR purification kit (Qiagen Valencia, CA), quantified using Tecan fluorometric methods (Tecan Group Mannedorf, Switzerland), normalized, and then pooled in preparation for Illumina MiSeq sequencing using the dual index 2 × 250 format V2 chemistry 500 cycles (Illumina Inc, La Jolla, CA) following the manufacturer’s protocol^32^.

### 16S analysis

Operational taxonomic units (OTUs) were generated *de novo* from raw sequence reads using the UPARSE pipeline^33^. Paired end (PE) reads were trimmed of adapter sequences, barcodes and primers prior to assembly. Sequences of low-quality, and singletons were discarded, and the remaining sequences were subjected to a dereplication step and abundances were determined. Chimera filtering of the sequences was completed during clustering while taxonomy was assigned to the OTUs with mothur^34^ using version 123 of the SILVA 16S ribosomal RNA database^35^ as the reference. OTUs and corresponding taxonomy assignment tables were generated and used in subsequent analyses. Downstream analyses were performed using the R statistical platform^36^. The OTU counts were normalized to the total number of reads per sample. Additionally, samples with less than 1,000 reads were excluded from the analysis. Dimensionality reduction was performed using a Principal Component Analysis (PCA) with the ‘prcomp’ function on the taxonomic abundance matrix. Major contributing OTUs to each principal component (PC), were determined by screening for the OTUs with the absolute value of the loading ≥ mean of loadings on the PC $\mp$4^37^. Association between the PCs and the metadata was assessed by fitting a generalized linear model (GLM), where PC is the response and predictors are the available clinical metadata.

### Longitudinal statistical analysis

Longitudinal data were modeled using the linear mixed-effects model^38^. To assess the association between microbiome diversity and caries status, we set the diversity index as the response and the caries group as the fixed effect term. To account for the two layers of correlation in the longitudinal data, i.e., (a) within twins and (b) within repeated measurements of each subject, *nested* random effects with subjects nested in twins were customized for the model. The linear mixed-effects model was performed using the R package ‘lme4’^39^ and the corresponding statistical test was conducted using the R package ‘lmerTest’^40,41^.

## Supplementary information

Supplementary Information.

## Data Availability

16S rRNA sequence reads have been deposited in the National Center for Biotechnology Information (NCBI) database under BioProject accession PRJNA383868, and under Sequence Read Archive (SRA) accessions SRR5467515 - SRR5467785 and SRR5467788 - SRR5468062.
