# Supplementary material for: Longitudinal Study of Oral Microbiome Variation in Twins
Source: Sci Rep. 2020 May 14;10:7954. doi: 10.1038/s41598-020-64747-1 (PMC7224172; doi:10.1038/s41598-020-64747-1)
Supplement: Supplementary file 1 — Supplementary Information. [file 41598_2020_64747_MOESM1_ESM.docx]

Longitudinal Study of Oral Microbiome Variation in Twins

Marcelo Freire^1,*,#^, Ahmed Moustafa^1,2,#^, Derek M. Harkins^3^, Manolito G. Torralba^1^, Yun Zhang^1^, Pamela Leong^4^, Richard Saffery^4^, Michelle Bockmann^5^, Claire Kuelbs^1^, Toby Hughes^5^, Jeffrey M. Craig^4^ & Karen E. Nelson^1,3^

^1^ Departments of Human Biology and Genomic Medicine, J. Craig Venter Institute, La Jolla, CA 92037, USA

^2^ Department of Biology, The American University in Cairo, New Cairo 11835, Egypt

^3^ Departments of Human Biology and Genomic Medicine, J. Craig Venter Institute, Rockville, MD 20850, USA

^4^ Murdoch Children’s Research Institute and Department of Pediatrics, University of Melbourne, Royal Children’s Hospital, Parkville, VIC 3052, Australia

^5^ Adelaide Dental School, The University of Adelaide, Adelaide, SA 5005, Australia

^#^ Contributed equally

**Running Title:** Longitudinal Caries Microbiome in Twins

* *Corresponding author*

Marcelo Freire, DDS, PhD, DMedSc

Associate Professor

Genomic Medicine and Infectious Diseases

4120, Capricorn Lane, 92037

La Jolla, CA

[mfreire@jcvi.org](mailto:mfreire@jcvi.org)

# Supplementary Material

## Supplementary Figure 1. Variance explained by the most relevant principal components of the oral microbiome abundances.


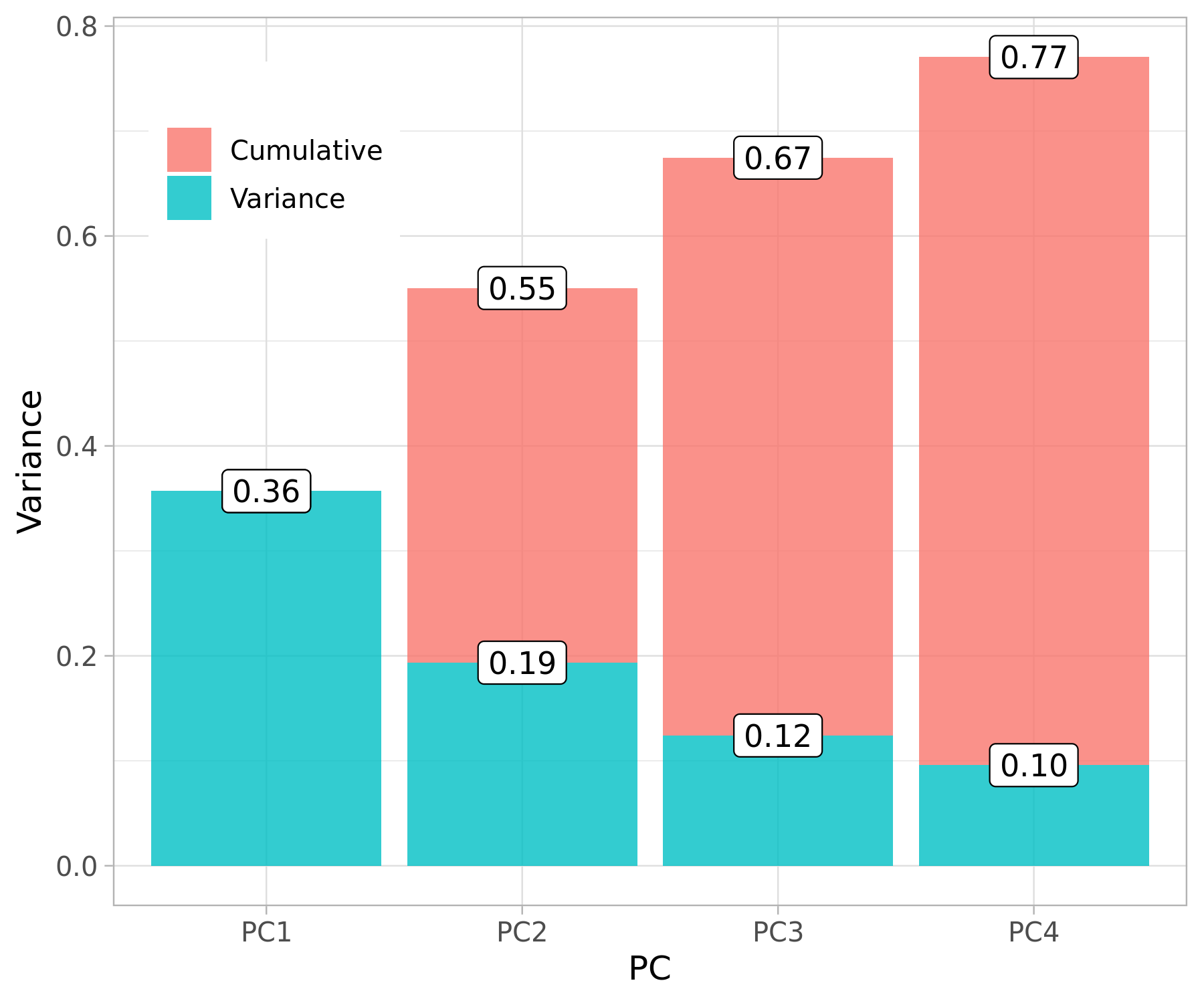


## Supplementary Figure 2. Abundance of major contributing OTUs against the age.

The x-axis is the age and the y-axis is the normalized taxonomic abundance. *P*-values of linear regression test are indicated under the name of the OTU. Significantly differentially abundant OTUs (*p*-value < 0.05) are indicated by “[sig]”. The blue line is the linear regression of the taxonomic abundances on the age.


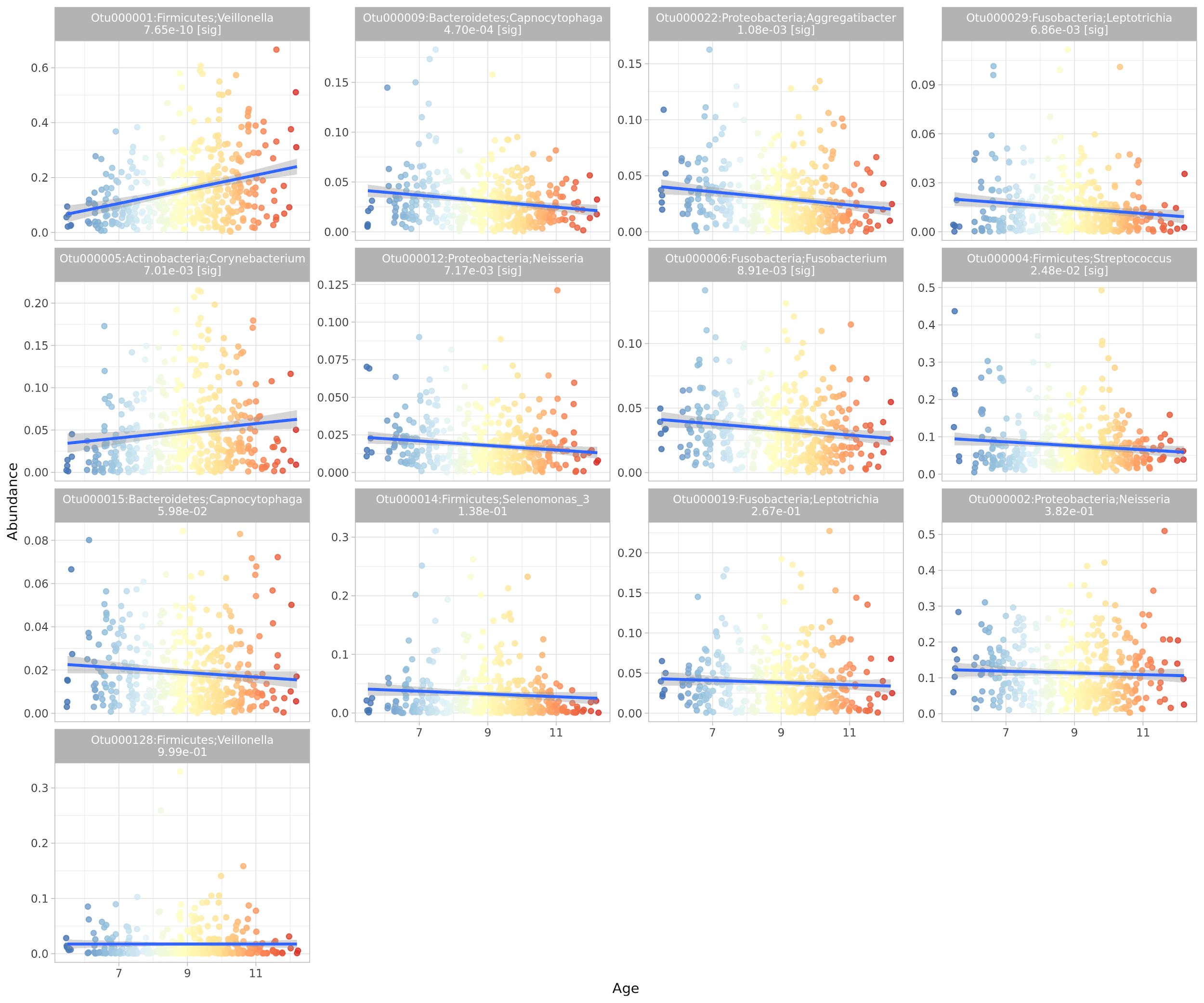


## Supplementary Figure 3. Abundance of major contributing OTUs against the visit.

The x-axis is the visit (V1; visit 1, V2; visit 2, and V3; visit 3) and the y-axis is the normalized taxonomic abundance. P-values of Kruskal test are indicated under the OTU. Significantly differentially abundant OTUs (p-value < 0.05) are indicated by “[sig]”. The blue line is the linear regression of the taxonomic abundances on the visit.


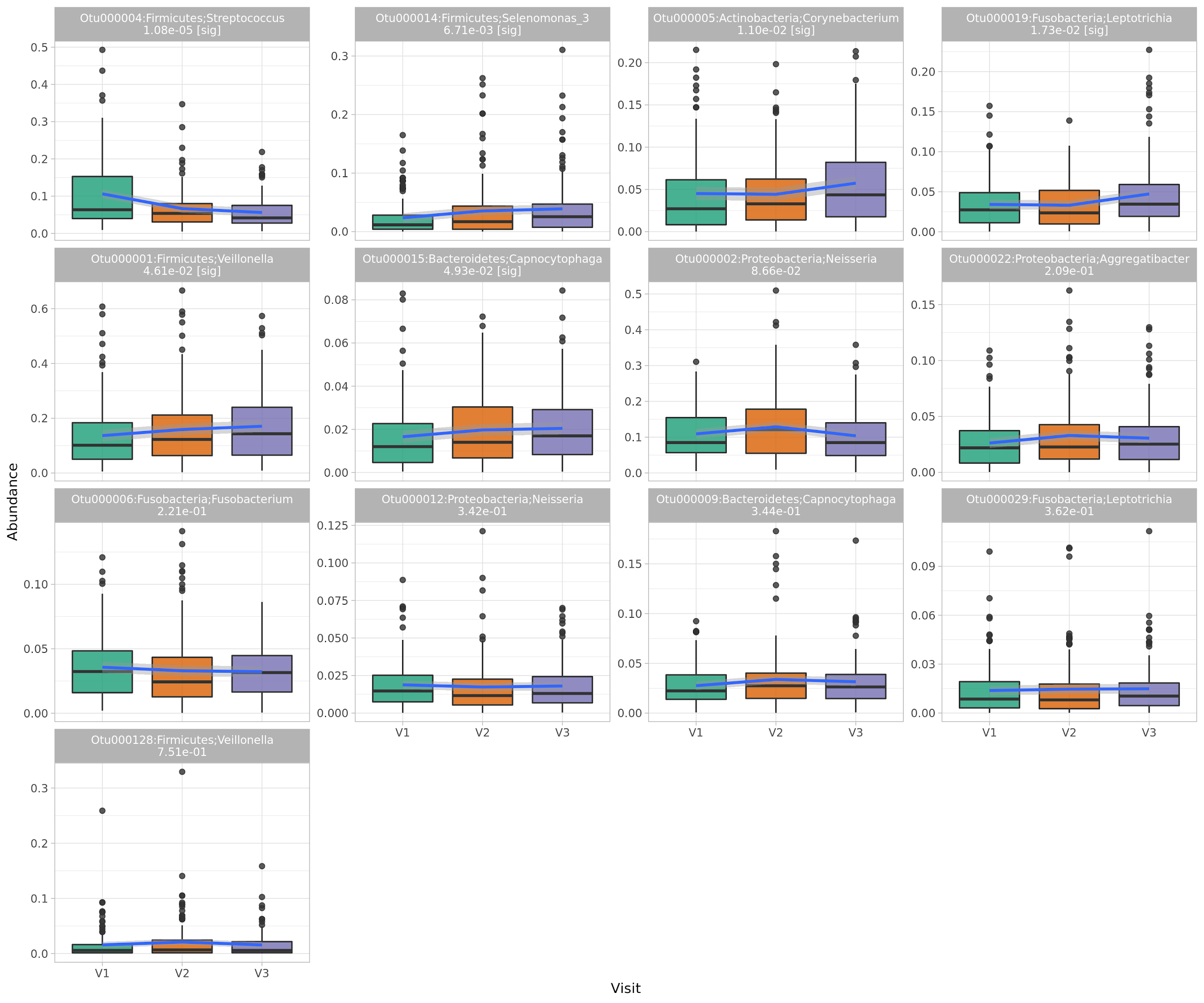


## Supplementary Figure 4. Abundance of major contributing OTUs against the age of starting brushing.

The x-axis is the age of brushing and the y-axis is the normalized taxonomic abundance. *P*-values of Kruskal test are indicated under the OTU. Significantly differentially abundant OTUs (*p*-value < 0.05) are indicated by “[sig]”. The blue line is the linear regression of the taxonomic abundances on the age of starting brushing.


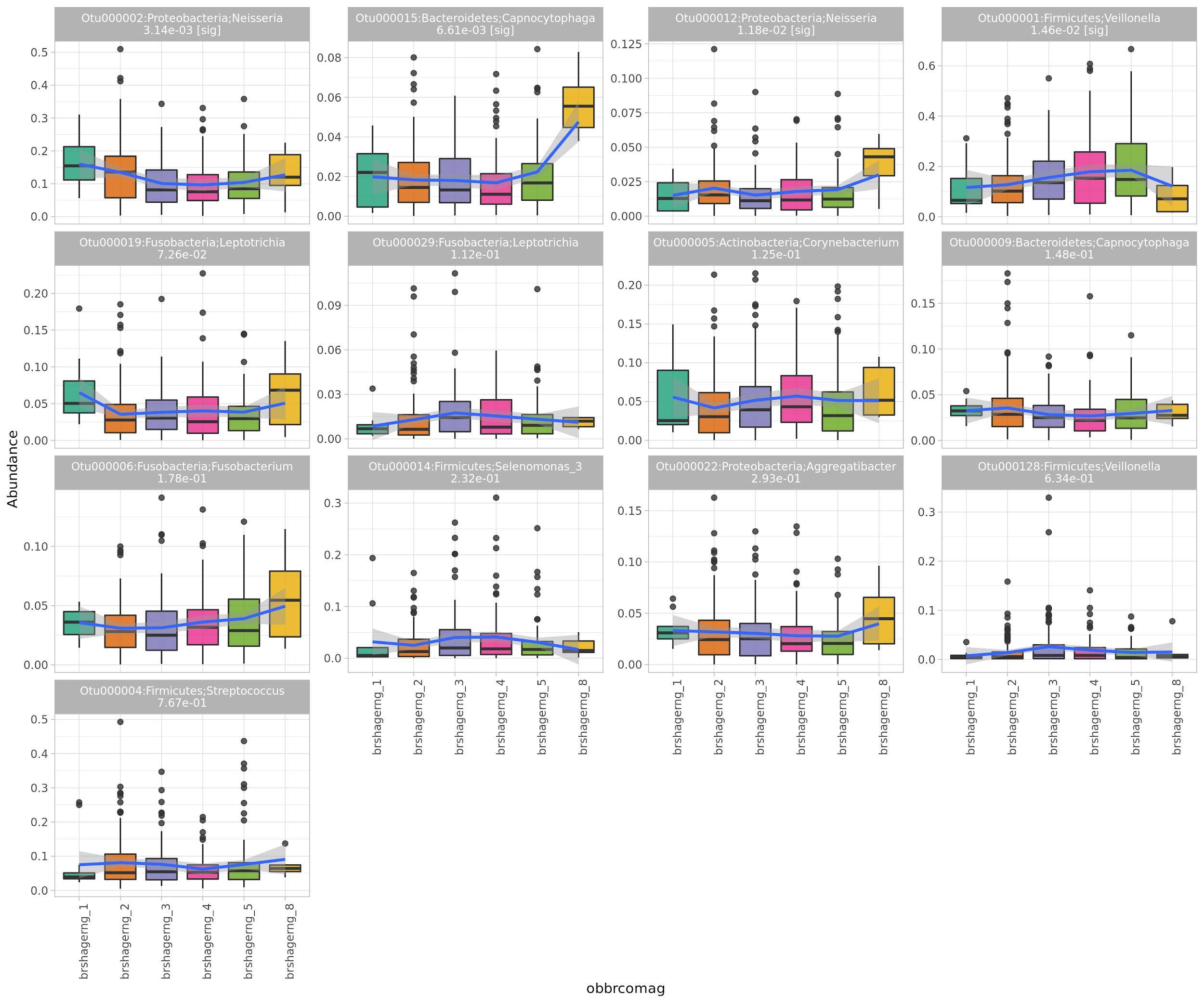


## Supplementary Figure 5. Abundance of PC1 major contributing OTUs against the rinsing habit.

The x-axis is the rinsing habit and the y-axis is the normalized taxonomic abundance. P-values of Kruskal test are indicated under the OTU. Significantly differentially abundant OTUs (*p*-value < 0.05) are indicated by “[sig]”. The blue line is the linear regression of the taxonomic abundances on the rinsing habits.


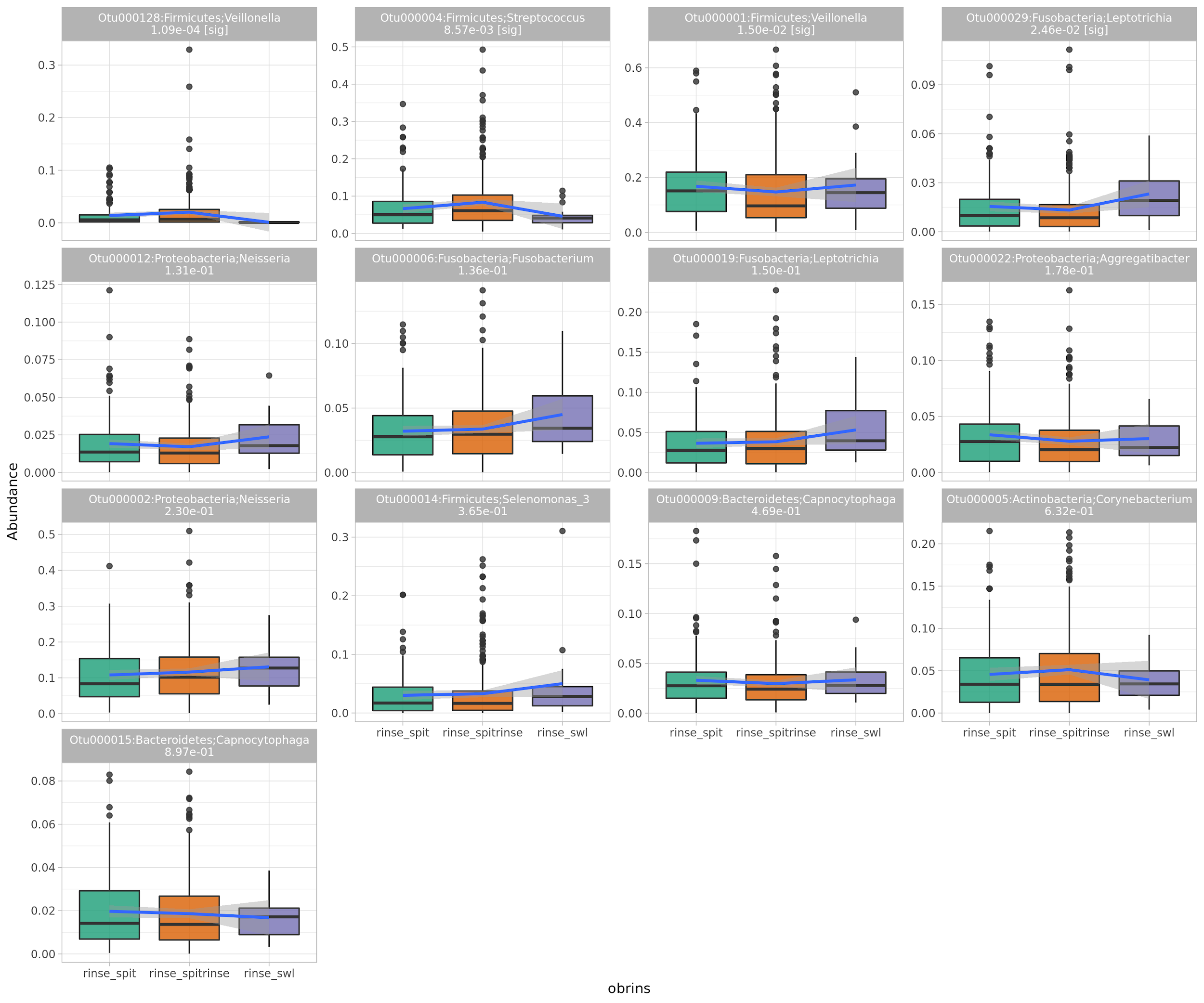


## Supplementary Figure 6. Environmental contributions to the oral microbiome overtime.

The y-axis is cross validated feature contributions (change of predicted probability due to metadata variables), x-axis is metadata variable value. Fitted line (LOO-kNN-gaussian) describes how well each variable effect is described by the metadata variable itself. R2 quantifies the goodness-of-fit of when visualizing the effect of the metadata variable effect on the predicted change main effect. The ages of the samples were grouped into three age groups (A; young, B; middle, and C; old). Panels with numeric labels 1-4 correspond to microbiome principal components PC1-PC4, respectively. Panels with letters A-D correspond to metadata variables, subject’s age, age of starting brushing, visit, and rinsing habits, respectively, where for example, A1 show the “age” contributing to microbiome “PC1”.


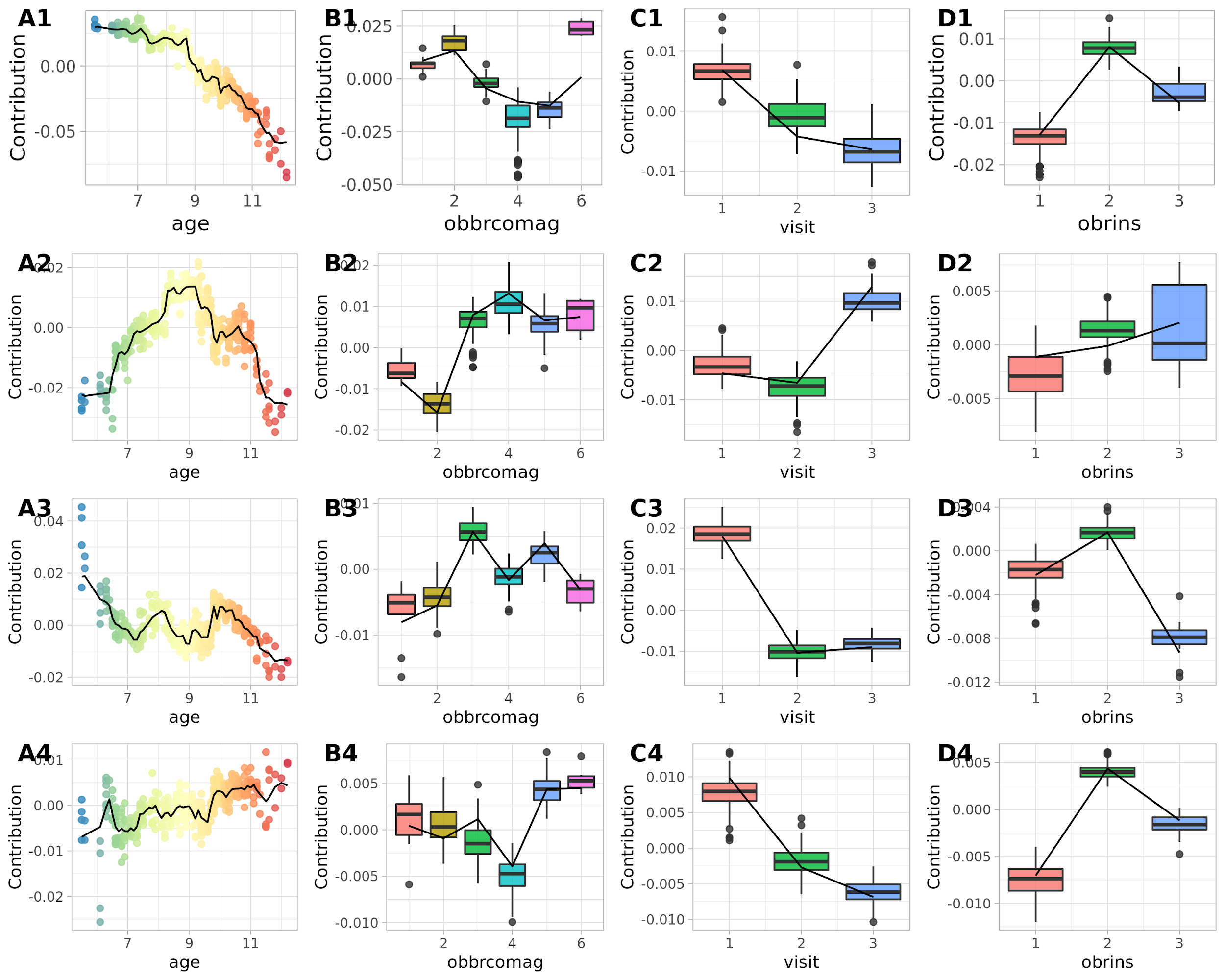


## Supplementary Figure 7. Oral microbiome distance by relationship and visit.

Dissimilarity was estimated using the Bray–Curtis index. same_family: monozygotic, dizygotic, and opposite-sex dizygotic twins; same_person: same individual across different samples. same_visit: the same visit i.e., V1, V2, or V3.


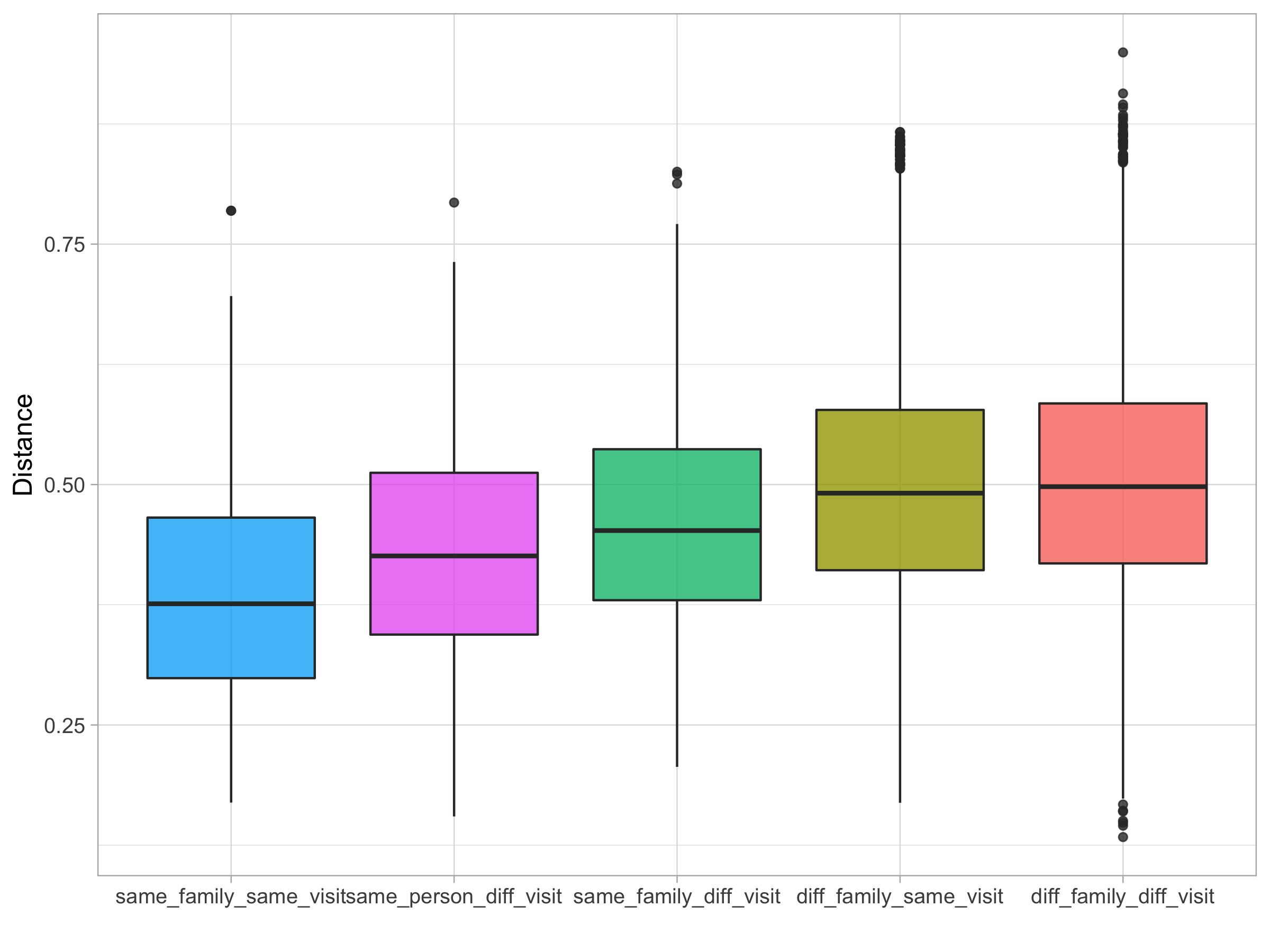


## Supplementary Figure 8. Oral microbiome distance between the visits. Dissimilarity was estimated using the Bray–Curtis index.

The boxplots show the distances between the microbiome of each person between the three visits.


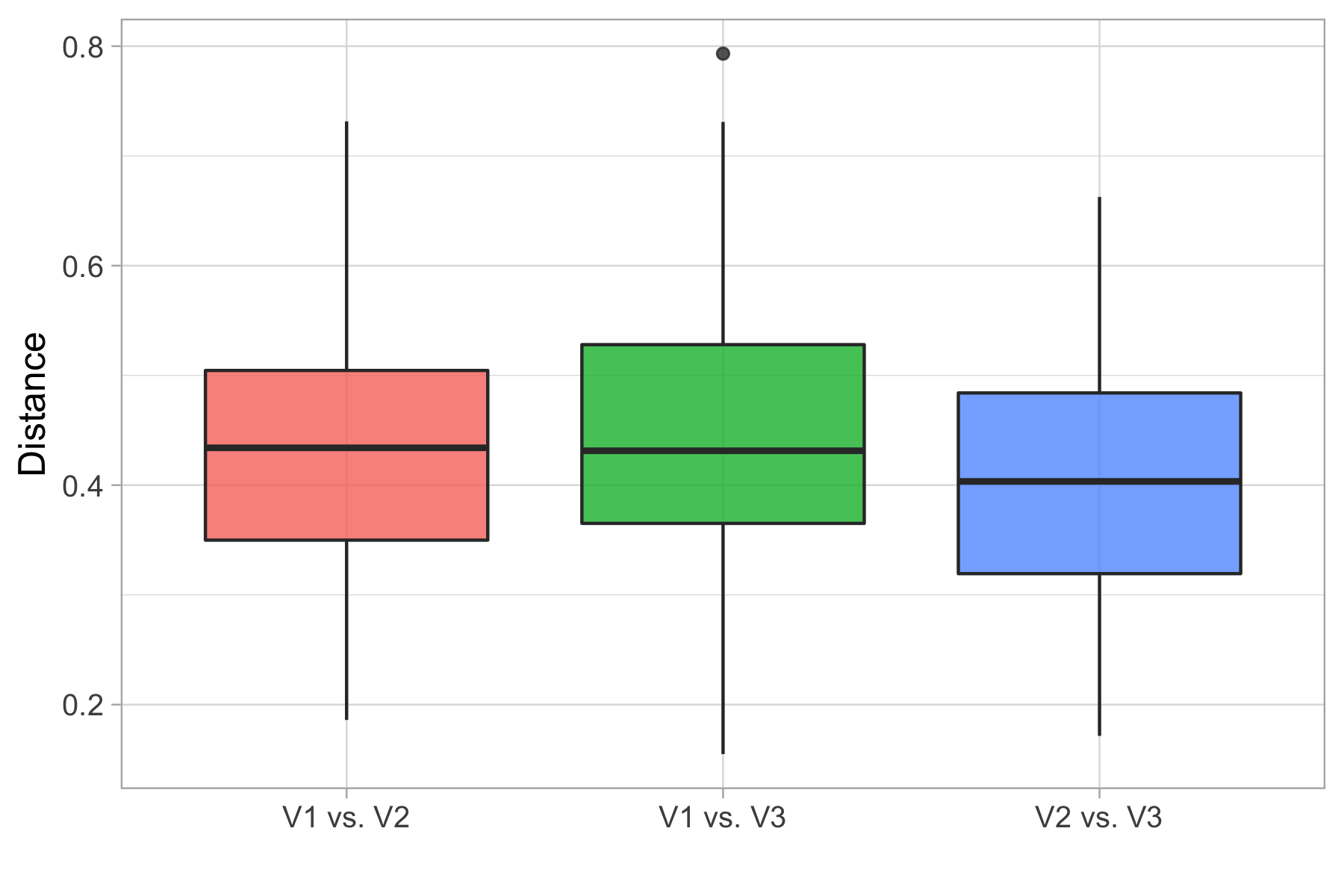


## Supplementary Figure 9. Longitudinal association of oral microbiome diversity and caries in enamel and dentin.

Each subject is represented by a line throughout the 3 visits (v1-v3). Caries status is indicated by color: blue (with caries) and red (without caries). P-values are the significance of testing oral microbiome diversity is different with or without caries using the linear mixed-effect model.


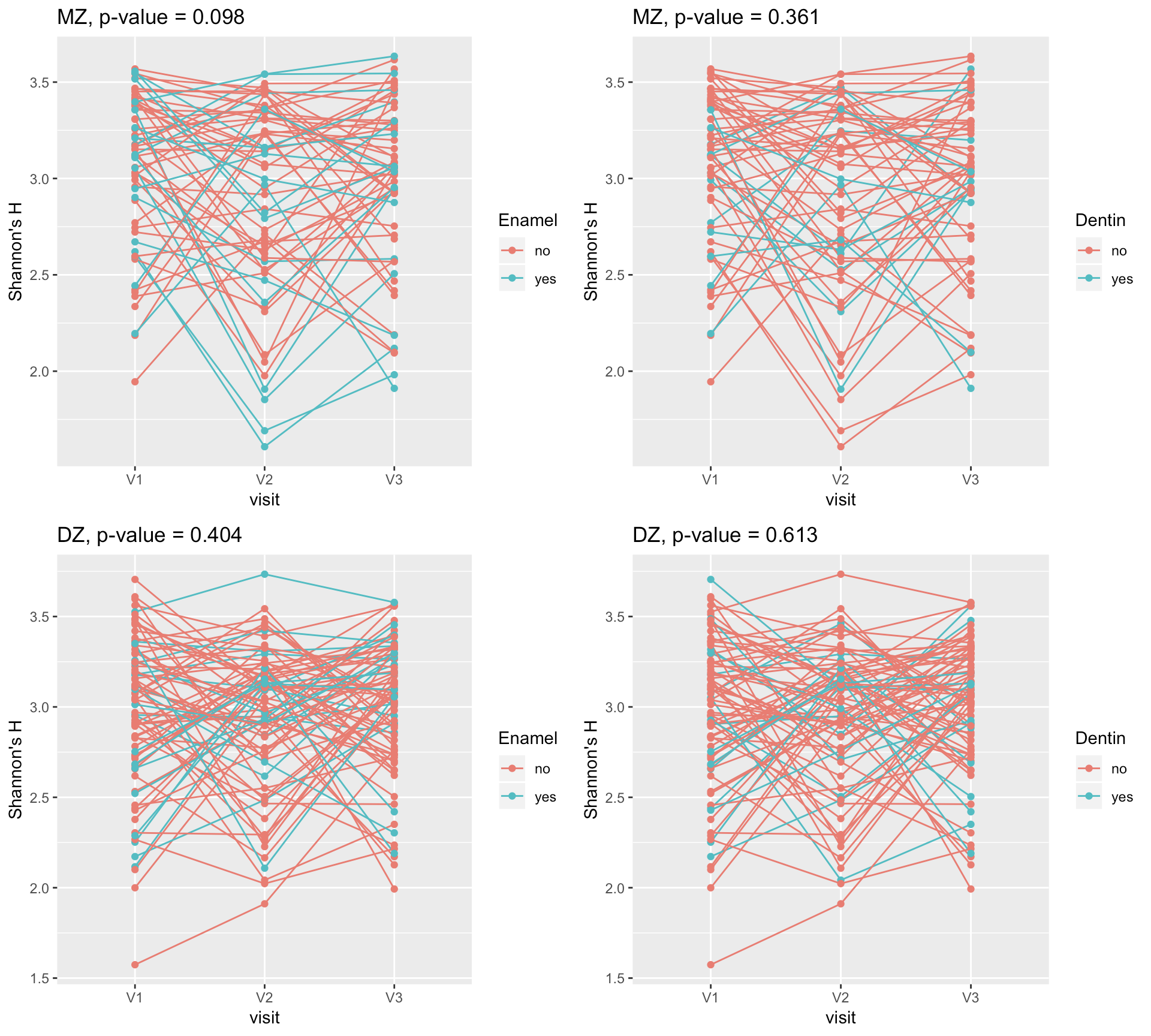


Supplementary Table 1. Glossary of variables, definitions, possible values, and questions asked on the questionnaire.

| **Variable** | **Possible values and definitions** | **Question asked on the questionnaire** |
| --- | --- | --- |
| sex | Sex of the twin  M = Male  F = Female | N/A (Not on questionnaire; Recorded by clinician) |
| zygosity | Zygosity of the twin  MZ = monozygotic  DZ = same sex dizygotic  OSDZ = opposite sex dizygotic | N/A (Not on questionnaire; Recorded by clinician) |
| visit | Timepoint tag    V1 = first visit, Visit 1  V2 = second visit, Visit 2  V3 = third visit, Visi t3 | N/A (Not on questionnaire; Recorded during  sample processing) |
| collection_date | Date of sample collection  DD-MMM-YY, day-Month-year | N/A (Not on questionnaire; Recorded by clinician) |
| age | Age of twins at the time of sampling  (in months) | N/A (Not on questionnaire) |
| dcsugcfr | Sugar added to fruit  sugcfr_nvr = never  sugcfr_smt = sometimes  sugcfr_alw = always  NA = data missing | ”Do you add sugars or sweeteners to the twins  stewed/cooked fruit?” |
| demuchfd | Relative food of the twins  muchfd_1 = Twin 1 eats a much greater amount of food  muchfd_2 = Twin 1 eats a somewhat greater amount of food  muchfd_3 = Twin 1 eats the same amount of food as Twin 2  muchfd_4 = Twin 2 eats a somewhat greater amount of food  muchfd_5 = Twin 2 eats a much greater amount of food  NA = data missing | ”In general, do your twins usually differ in how  much they eat?” |
| desug | Sugar added to food  sug_nvr = never  sug_smt = sometimes  sug_alw = always  NA = data missing | “Is sugar added to the twins food/drinks (other than  deserts, puddings etc.)” |
| devarfd | Variety of food fed to the twin  vartyfd_1 = Twin 1 eats a much greater variety of food  vartyfd_2 = Twin 1 eats a somewhat greater variety of food  vartyfd_3 = Twin 1 eats the same variety of food as Twin 2  vartyfd_4 = Twin 2 eats a somewhat greater variety of food  vartyfd_5 = Twin 2 eats a much greater variety of food  NA = data missing | “In general, do your twins usually differ in  what they eat?” |
| hcclap | Presence or absence of cleft palate in twin  cleft_yes = presence of cleft palate  cleft_no = absence of cleft palate  NA = data missing | ”Do either of your twins have a cleft lip and/or  palate, or other craniofacial condition?” |
| heab | Antibiotic use by twin  ab_yes = yes  ab_no = no  ab_dnr = do not recall  NA = data missing | ”Were either of your twins given antibiotics during  the first week following their birth?” |
| heabmo | Antibiotic use by mother during pregnancy  abmom_yes = yes  abmom_no = no  abmom_dnr = do not recall  NA = data missing | ”Were you given antibiotics during your pregnancy  with the twins?” |
| obthbr | Is the twin currently brushing?  brsh_yes = yes  brsh_no = no  NA = data missing | ”Have your twins started tooth brushing?” |
| obbrcomag | Age at which twin began brushing.  brshagerng_1 = <6 months of age  brshagerng_2 = 6 - 12 months ofage  brshagerng_3 = 1 - 1.5 years of age  brshagerng_4 = 1.5 - 2 years of age  brshagerng_5 = 2 - 2.5 years of age  brshagerng_6 = 3 - 4 years of age  brshagerng_7 = 4 - 5 years of age  brshagerng_8 = 5 - 6 years of age  brshagerng_9 = older than 6 years of age  NA = data missing | ”If yes, approximately how old were they when  brushing started?” |
| obbrfrq | Brushing frequency  brshfreq_1 = twice or more daily  brshfreq_2 = once daily  brshfreq_3 = every 2 - 4 days  brshfreq_4 = weekly  brshfreq_5 = Not at all/rarely  NA = data missing | ”How often are your twins' teeth brushed?” |
| obtbrush | Brush use. Is a brush used for hygiene?  tthbrsh_yes = yes, brush is used  tthbrsh_no = no, brush not used  NA = data missing | ”Is a toothbrush always used to clean your  twins' teeth?” |
| obtpast | Toothpaste use. Is toothpaste used during brushing?  tthpst_yes = yes, toothpaste is used  tthpst_no = no, toothpaste not used  NA | ”Is toothpaste used to brush your twins' teeth?” |
| obtpasty | Toothpaste type used  tthpstTyp_adltF = adult, fluoride  tthpstTyp_chldF = child with fluoride  tthpstTyp_adlt = adult, no fluoride  tthpstTyp_othr = other  NA = data missing | ”If yes, what type of toothpaste do you use  for the twins?” |
| obrins | Rinsing habits    rinse_spitrinse = spit and rinse  rinse_spit = spit and no rinse  rinse_swl = swallow  NA = data missing | “When your twins brush their teeth do,  they usually…” |
| obpasamt | Toothpaste amount. How much toothpaste is used for brushing?  thtpstamt_full = brush full  thtpstamt_pea = pea-sized  thtpstamt_smr = smear  NA | How much toothpaste is commonly used on  the twins' toothbrushes? |
| dencarenam | Untreated enamel-only caries  carenam_yes = presence of caries in enamel  carenam_no = absence of caries in enamel  NA = data missing | N/A (Not on questionnaire; Determined by clinician) |
| dencarden | Untreated caries into dentin  cardent_yes = presence of caries in dentin  cardent_no = absence of caries in dentin  NA = data missing | N/A (Not on questionnaire; Determined by clinician) |
| dencartreat | Any restored/missing teeth due to caries  cartreat_yes = yes  cartreat_no = no  NA = data missing | N/A (Not on questionnaire; Determined by clinician) |
| discordant | Twin 1 and twin 2 discordant for caries experience  discord_yes = Twins discordant  discord_no = Twins not discordant  NA | N/A (Not on questionnaire; Determined by clinician) |
